# The Effect of Functionalized SEBS on the Properties of PP/SEBS Blends

**DOI:** 10.3390/polym15183696

**Published:** 2023-09-08

**Authors:** Lixin Song, Fei Cong, Wei Wang, Jiannan Ren, Weihan Chi, Bing Yang, Qian Zhang, Yongchao Li, Xianliang Li, Yuanxia Wang

**Affiliations:** 1 Polymer High Functional Film Engineering Research Center of Liaoning Province, Shenyang University of Chemical Technology, Shenyang 110142, China; 18841740139@163.com (F.C.);; 2 College of Materials Science and Engineering, Shenyang University of Chemical Technology, Shenyang 110142, China; 3 BatteroTech Co., Ltd., Shanghai 201417, China; 15541954253@163.com; 4 AVIC Shenyang Aircraft Corporation, Shenyang 110850, China

**Keywords:** PP, SEBS, SEBS-g-(GMA-co-St) graft copolymer, compatibility, mechanical properties

## Abstract

Styrene (St) was used as comonomer and glycidyl methacrylate (GMA) as grafting monomer to prepare SEBS-g-(GMA-co-St) graft copolymers via melt grafting. Then, the graft copolymers were employed as a compatibilizer for melt blending polypropylene (PP) and hydrogenated styrene-butadiene-styrene (SEBS) triblock copolymers. The effects of the amount of GMA in the graft copolymers on thermal properties, rheology, crystallization, optical and mechanical properties, and microstructure of the blends were investigated. The results show that GMA and St were successfully grafted onto SEBS. The GMA amount in the graft copolymer significantly influenced the comprehensive properties of PP/SEBS/SEBS-g-(GMA-co-St) blends. The epoxy groups of GMA reacted with PP and SEBS, forming interfacial chemical bonds, thereby enhancing the compatibility between PP and SEBS to varying extents. After introducing SEBS-g-(GMA-co-St) into PP/SEBS blends, crystallinity decreased, crystal size increased while transmittance remained above 91% with rising GMA amount in the graft copolymers, indicating excellent optical properties. Notched impact strength and elongation at break of the blends showed a trend of first increasing and then decreasing with increased amounts of GMA in the graft copolymers. When the amount of GMA in the graft copolymers was 3 wt%, the blends exhibited optimal toughness with notched impact strength and elongation at break of 30,165.82 J/m^2^ and 1445.40%, respectively. This was attributed to the tightest dispersion interface adhesion and maximum matrix plastic deformation, consistent with the mechanical performance results.

## 1. Introduction

Polypropylene (PP) is one of the most widely used general-purpose plastics, characterized by its low density, low cost, good heat resistance, and balanced mechanical properties [1,2,3,4,5,6,7]. As a result, PP is extensively used in industries such as home appliances, automotive, and construction. However, the high shrinkage rate and relatively low notched impact strength of PP have greatly limited its further promotion and application [8,9,10]. To eliminate this disadvantage, PP can be blended with a thermoplastic elastomer to improve the toughness of PP [11,12,13].

Previous studies have found [14,15,16] that the properties of PP and hydrogenated styrene-butadiene-styrene block copolymer (SEBS) complement each other well. SEBS is a new type of polymer with styrene-ethylene-butadiene-styrene as its block. It has excellent mechanical properties, thermal properties, oil resistance, aging resistance, electrical properties, chemical properties, and surface properties [17,18,19,20]. The addition of SEBS elastomer to the PP matrix not only improves its mechanical properties but also its processing properties [21,22]. However, due to the low compatibility between PP and SEBS, the resulting blend cannot meet the demand of practical applications in terms of mechanical properties. Therefore, it is necessary to enhance the compatibility between PP and SEBS to improve the mechanical properties of the blend [23,24,25,26]. This further improves the cost-effectiveness of the compatibilized blends, greatly expanding its application scope, such as in washing machine parts, refrigerator parts, rotary boxes, seats, and automotive parts, among others.

To improve the compatibility between the elastomer and PP, the elastomer can be functionalized before being melt extruded with PP. The interfacial bonding strength is reinforced through in situ grafting reactions to achieve better toughening effects [27,28,29]. Maleic-anhydride (MAH)- [30,31,32] and glycidyl-methacrylate (GMA)-grafted [33,34,35,36,37] polymers are typically used as reactive compatibilizers, both of which can improve compatibility by forming strong chemical bonds between the blends. Yin et al. [38] used PP-g-GMA as a compatibilizer for melt blending with PP/polycarbonate (PC). The study found that the compatibility between PP and PC was enhanced by forming in situ copolymers during the blending process. The tensile strength and impact strength of the PP/PC/PP-g-GMA blend increased by 90% and 67%, respectively, compared to the PP/PC blend. Martins et al. [39] grafted MAH onto PP to obtain PP-g-MAH graft copolymer, which was melt blended with thermoplastic starch (TPS) to produce a PP/TPS/PP-g-MAH blend. The addition of the graft copolymer enhanced the interfacial interaction between PP and TPS, significantly improved the toughness of the blend, with a 194% increase in the elongation at break. Niu et al. [40] used PP-g-MAH to compatibilize PP and hemp fiber (HF) composites. The results showed that the introduction of PP-g-MAH enhanced the interaction between PP/HF, significantly improving the impact strength and elongation at break of the composite material. Cartier et al. [41] used PP-g-GMA graft copolymer to compatibilize PP and polybutylene terephthalate (PBT). The study found that the elongation at break of the PP/PBT blend increased from 50% to 260%, and the toughness was significantly improved after the addition of PP-g-GMA graft copolymer.

The main structure of MAH consists of anhydride groups and double bonds between two carbon atoms, which can undergo free radical polymerization in the presence of initiators [42]. Recent studies have shown that MAH grafting can be optimized, but the efficiency is still low [43,44]. Shen et al. [45] pointed out that the volatile MAH monomer has an irritating odor and is harmful to the body. Lyu et al. [46] pointed out that due to the low grafting rate, volatility, and irritation of MAH, the harm of volatile MAH gases to the body under melt grafting conditions are especially pronounced. In view of the disadvantages of MAH such as low grafting efficiency, easy volatilization at high temperatures, high irritation to the body, and easy corrosion of equipment, GMA with high boiling point, low toxicity, and relatively high grafting rate has replaced MAH and is widely used in the functional modification of elastomers [47]. Its grafted products play an extremely important role in the compatibilization modification of plastics. During the melt blending process, the epoxy groups in GMA and polyester hydroxyl groups form copolymers at the interface between the two phases through nucleophilic substitution reactions [48], which reduces the interfacial tension and enhances the bonding strength between the two phases. Therefore, GMA was chosen as the graft monomer for SEBS in this study.

Based on the above analysis, this paper utilizes dicumyl peroxide (DCP) as the initiator, GMA as the graft monomer, and styrene (St) as the comonomer. The SEBS-g-(GMA-co-St) graft copolymer is prepared by using the free radical melt grafting technique. Subsequently, the prepared graft copolymer is melt-blended with PP and SEBS resin to prepare the PP/SEBS/SEBS-g-(GMA-co-St) blend. The effects of varying the amount of GMA in the graft copolymer on the grafting rate and grafting efficiency were investigated. Furthermore, the impact on the blend’s crystallization ability, thermal properties, rheological properties, optical properties, mechanical properties, and microstructure was explored.

## 2. Material and Methods

### 2.1. Materials

Polypropylene (PP), grade: RP340R, density: 0.910 g/cm^3^, melt flow rate: 20.0–24.0 g/min, supplied by China Lanzhou Petrochemical Company (Lanzhou, China). 

Hydrogenated styrene-butadiene block copolymer (SEBS), grade: 1651, density: 0.910 g/cm^3^, polypropylene content: 30.0–33.0%, supplied by US KOTEN Corporation (Houston, TX, USA).

Glycidyl methacrylate (GMA), analytical grade, supplied by US Dow Chemical Company (Midland, MI, USA). 

Styrene (St), chemically pure, a colorless and transparent liquid with 99% content and 0.909 g/mL density, supplied by Tianjin Damao Chemical Reagent Plant (Tianjin, China).

Dicumyl peroxide (DCP), chemically pure, supplied by Chengdu Best Reagent Co., Ltd. (Chengdu, China).

Acetone, analytical grade, density: 0.789 g/cm^3^, supplied by Changchun Chemical (Jiangsu) Co., Ltd. (Suzhou, China).

### 2.2. Preparation of the Graft Copolymers

The SEBS was dried at 60 °C for 12 h in a vacuum oven. Then, DCP was dissolved in a mixture solution of GMA and St. After complete dissolution, it was uniformly mixed with SEBS according to a certain mass ratio. The melting graft reaction was carried out in a torque rheometer, and the resulting product was cooled for 24 h and cut into pellets for later use. The compounding conditions were as follows: temperature of 200 °C, rotational speed of 70 r/min, and time of 5 min. The grafted product was denoted as SEBS-g-(GMA-co-St), and the specific formula is shown in Table 1 [49].

### 2.3. Purification of the Graft Copolymer and Determination of Grafting Degree

#### 2.3.1. Purification of Graft Copolymers

Approximately 2 g of the graft copolymer was weighed and refluxed in 25 mL of xylene for 1 h. Then, at room temperature, the unreacted monomers were removed via filtration using acetone as the solvent with a vacuum filtration apparatus [50]. The purified samples were dried under vacuum at 70 °C for 12 h.

#### 2.3.2. Determination of Grafting Degree

Accurately weigh 0.2 g of the purified graft sample. Reflux the sample in 50 mL of xylene and cool slightly. Transfer the sample to a 15 mL solution of 0.05 mol/L HCl-isopropanol and reflux for 2 h. Use phenolphthalein as an indicator and titrate with 0.05 mol/L NaOH-ethanol standard solution until the endpoint.

The grafting degree (GD) of GMA is calculated according to Equation (1):

(1) $$\mathrm{GD}(\%)=\frac{N(V_{0}-V)\times 142.15}{1000W}\times 100$$

where, *N* is the concentration of NaOH-methanol standard solution in mol/L. *V*_0_ is the volume of NaOH-methanol standard solution consumed by pure SEBS during titration in ml. *V* is the volume of NaOH-methanol standard solution consumed during titration in ml. *W* is the mass of the added purified sample in g.

### 2.4. Preparation of the Blends

After drying, PP, SEBS, and the SEBS-g-(GMA-co-St) graft copolymers were melt blended in a torque rheometer according to a certain mass ratio (rotation speed of 70 r/min, compounding temperature of 200 °C, compounding time of 5 min). It has been found in studies that when the mass ratio of PP to SEBS is 80/20, the toughness of the blend material is optimal [51]; therefore, this ratio was adopted for the research in this paper. The specific composition of the blend is shown in Table 2. The blended mixture was then injection molded into standard specimens for impact and tensile testing using a micro-injection molding machine. The specimens were conditioned at 25 °C for 24 h before testing.

### 2.5. Characterization

Fourier Transformation Infrared Spectroscopy (FTIR) Analysis: The purified and dried PP/SEBS/SEBS-g-(GMA-co-St) blends were molded into a film on a plate vulcanizer (400 × 400 × 2, Qingdao Yadong Rubber Machinery Co., Ltd., Qingdao, China) and analyzed via FTIR. FTIR spectra were recorded on a NEXUS 470 PC instrument. The infrared tests were performed in the scanning range of 5000~500 cm^−1^, with a resolution of 0.5 cm^−1^, a wavenumber accuracy of 0.01 cm^−1^, and a scanning rate of 75 scans/s.

^1^H-Nuclear Magnetic Resonance (^1^H-NMR) Analysis: Accurately weigh approximately 15 mg of the PP/SEBS/SEBS-g-(GMA-co-St) blends. Use Deuterated chloroform (CDCl_3_) as the solvent and tetramethylsilane (TMS) as the internal standard. The analysis is conducted using a Bruker Ascend 500 III nuclear magnetic resonance (NMR) spectrometer with 128 scans.

Dynamic mechanical analysis (DMA): The dynamic mechanical thermal analysis of the sample was tested with a DMA tester (PerkinElmer, Diamond DMA/DMS6100, Wellesley, USA). In the tension mode, the temperature was raised from −100 °C to 150 °C at a heating rate of 3 °C/min at a frequency of 1 Hz.

Differential scanning calorimetry (DSC) analysis: The crystallization and melting behavior of the samples were characterized using differential scanning calorimetry (DSC, TA Q200, TA, New Castle, DE, USA). Approximately 5 mg of the PP/SEBS/SEBS-g-(GMA-co-St) mixture was loaded into a crucible. The temperature was ramped from 0 °C to 200 °C at a rate of 10 °C/min and held for 5 min to eliminate any thermal history. Then, it was cooled back to 0 °C at the same rate. The airflow rate was set to 2.5 L/min.

The crystallinity can be calculated using Equation (2):

(2) $$X_{c}=\frac{∆H_{m}}{∆H_{m}^{0}(1-\varphi )}$$

In the formula, X_c_ is the crystallinity of the sample, $∆H_{m}$ is the heat released during sample melting, $∆H_{0}$ is the melting enthalpy with 100% crystallinity for PP (209 J/g) [52], and *φ* is the content of SEBS.

Wide-angle X-ray diffraction(WAXD)analysis: WAXD analysis was performed on a Bruker D8 ADVANCE diffractometer at room temperature, using Cu Kα radiation (λ = 1.5406 Å) operating at 40 kV and 40 mA. The sample was measured in the range of 5° to 60° with a scanning rate of 2°/min. 

Thermogravimetric analysis (TGA): Thermogravimetric analysis (TGA, STA449C, NETZSCH, Selb, Germany) was performed under flowing nitrogen (100 mL/min) at a heating rate of 10 °C/min. Approximately 15 mg of PP/SEBS/SEBS-g-(GMA-co-St) blends were heated from room temperature to 600 °C.

Vicat softening temperature testing:The Vicat softening temperature test (XWB-300C, Chengde Testing Machine Co., Ltd., Chengde, China) was conducted according to the GB/T 1633-2000 standard. A sample block was placed in the test area, and a weight of 1.02 kg was applied as the load. The temperature was increased at a rate of 50 °C/h, and the temperature at which the needle penetrated the sample by 1 mm was recorded.

Melt flow rate (MFR) analysis:The MFR was determined via a melt flow rate instrument (GT-7100-MH, Gotweil Scientific Instruments Co., Ltd., Qingdao, China) at 200 °C with a load of 2.16 kg (test standard was ASTM D1238-2013); MFR was calculated according to Equation (3):

(3) $$\mathrm{MFR}=m\times \frac{600}{t}$$

In the formula, *m* represents the mass of the test specimen in grams, and *t* represents the cutting time of the extrudate in seconds.

Rheological property analysis: The rheological measurements of the mixture were conducted using a Dynamic Mechanical Analyzer (DHR-2) from TA Instruments, located in New Castle, DE, USA. The frequency sweep of the PP/SEBS/SEBS-g-(GMA-co-St) mixture was performed under nitrogen gas using a 25 mm parallel plate geometry. The gap distance between the parallel plates was set to 0.8 mm. The thickness of the sheet-like mixture was approximately 1.0 mm. The angular frequency range used in the test was 0.1–100 rad/s, with a shear strain of 0.5%. The temperature is set to 235 °C.

Optical property analysis: The PP/SEBS/SEBS-g-(GMA-co-St) mixture was compressed into a thin film of approximately 80 µm thickness using a flat press. The film was then subjected to haze and transmittance testing using a CS-700 haze meter (Hangzhou Caipu Technology Co., Ltd., Hangzhou, China).

Mechanical property analysis: According to GB/T1843/1-A, Izod notch impact strength was determined with a GT-7045-MD impact tester (Songshu Instrument Co., Ltd., Dongguan, China), the pendulum used was 2.750 J. The tensile properties were measured according to GB/T1040-1BA using a tensile testing machine (Instron 3365, Instron, Boston, MA, USA) at a crosshead speed of 25 mm/min. The test was performed at room temperature, and the average values of at least five tests were reported

Scanning electron microscopy (SEM) analysis: The fractured surface of the PP/SEBS/SEBS-g-(GMA-co-St) mixture was coated with a thin layer of gold using a sputter coater. Then, it was observed using a scanning electron microscope (JSM-5600LV) from JEOL, located in Tokyo, Japan.

## 3. Results and Discussion

### 3.1. FTIR and ^1^H-NMR Analysis

**Figure 1:**
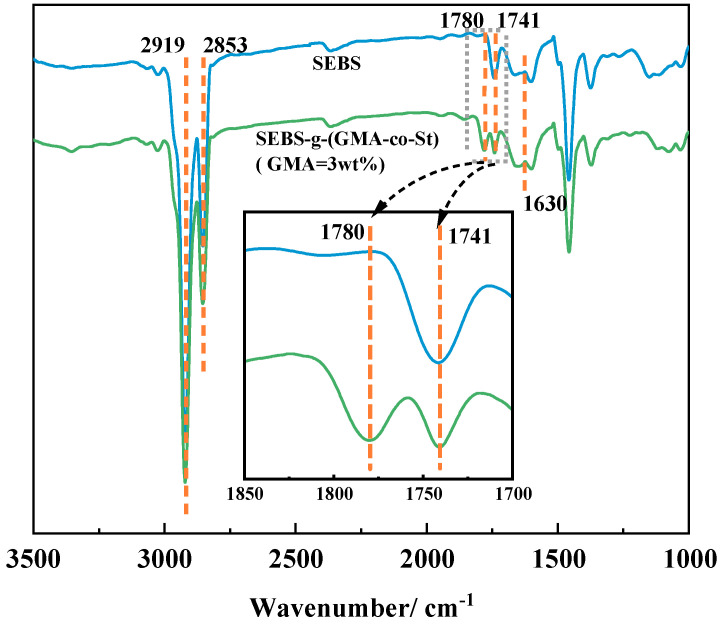
FTIR spectra of SEBS (blue line) and SEBS-g-(GMA-co-St) (GMA = 3 wt%) graft copolymer (green line).

To verify whether a grafting reaction has occurred between SEBS and GMA, we conducted infrared spectroscopy characterizations on both SEBS and the purified SEBS-g-(GMA-co-St) graft copolymer, with the test results shown in Figure 1. As can be seen from Figure 1, both the infrared spectra of SEBS and the graft copolymer exhibit signal peaks at 2919 cm^−1^ and 2853 cm^−1^, which are the absorption peaks of CH aliphatics in the SEBS backbone [53]; a signal peak appears at 1741 cm^−1^, which is the absorption peak of the aromatic ring in the SEBS backbone. By comparing the infrared spectra of SEBS and the SEBS-g-(GMA-co-St) graft copolymer, it is evident that the overall trends of the two spectra are roughly the same. The difference is that the graft copolymer containing GMA has a new absorption peak appearing at 1780 cm^−1^, which is caused by the carbonyl stretching vibration in GMA [54]. Moreover, the characteristic signal peak of the C=C functional group (around 1630 cm^−1^) does not appear in the spectrum of the graft copolymer, indicating that the GMA monomer has been completely removed during the purification process of the graft copolymer. This evidence supports that a grafting reaction occurred between SEBS and GMA during the melting process, resulting in the SEBS-g-(GMA-co-St) graft copolymer in the extrudate.

**Figure 2:**
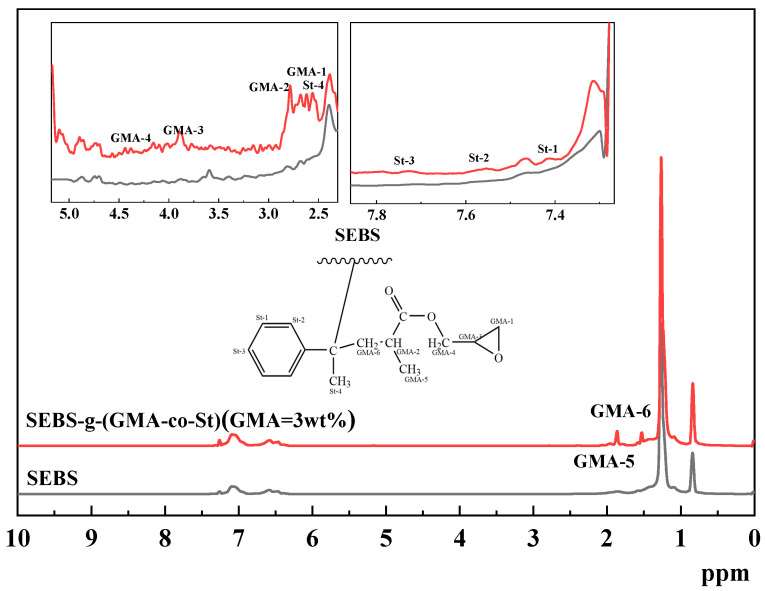
^1^H-NMR spectra of SEBS (black line) and SEBS-g-(GMA-co-St) (GMA = 3 wt%) graft copolymer (red line).

To further verify whether a grafting reaction occurred between SEBS and GMA, this study characterized both SEBS and the purified SEBS-g-(GMA-co-St) graft copolymer using nuclear magnetic resonance (^1^H-NMR). The test results are shown in Figure 2. As can be seen from Figure 2, there are three common signal peaks at 7.2, 6.5, and 1.3 ppm in the ^1^H-NMR spectra of both SEBS and SEBS-g-(GMA-co-St), which indicates that these spectra are based on SEBS. By enlarging the regions of 5.1–2.3 ppm and 7.8–7.2 ppm, four signal peaks at 7.73, 7.56, 7.41, and 2.55 ppm appear in the ^1^H-NMR spectrum of the SEBS-g-(GMA-co-St) graft copolymer, which are generally attributed to the signal peaks of St. Furthermore, five signal peaks appear at 4.39, 3.87, 2.78, 2.67, 1.85, and 1.53 ppm, which are generally attributed to the signal peaks of GMA [55,56,57]. This indicates that GMA has successfully grafted onto the SEBS macromolecular chain under the promotion of St.

**Figure 3:**
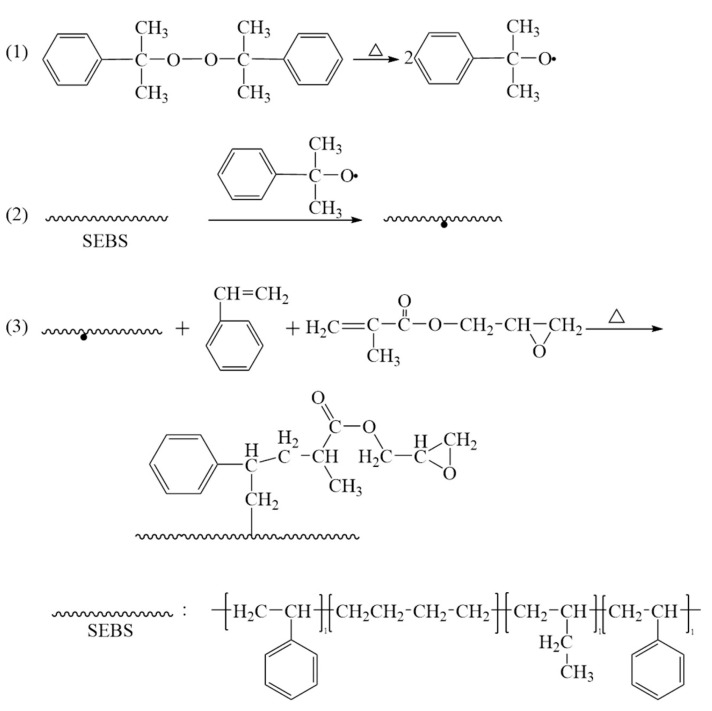
Reaction mechanism for preparing SEBS-g-(GMA-co-St) graft copolymer ((1) the initiator splits into two free radicals, (2) the free radical initiates SEBS with a reaction site, and (3) the grafting reaction).

In summary, with the promotion of St, GMA has successfully undergone grafting reaction and incorporated epoxy groups into the macromolecular chains of SEBS. This will facilitate the formation of chemical bonds between PP and SEBS, thereby improving the compatibility of PP/SEBS blends. The reaction route is shown in Figure 3.

### 3.2. Characterization of the Graft Copolymers

The grafting degree (GD) and grafting efficiency (GE) are two important indicators to measure the grafting reaction of polymers. In the experiments, the amount of DCP was kept constant, and the effect of the added amount of GMA in the grafted copolymer on the GD and GE of the SEBS-g-(GMA-co-St) grafted copolymer was examined. Specific data can be seen in Table 3.

The ratio of each pair of monomers in a specific copolymer can be determined through *Q*-*e* Equations (4) and (5) [58]. When the *Q* values of two monomers are close, they are more likely to undergo copolymerization. As shown in Table 4, the *Q* and *e* values of GMA and St are similar [56,59,60].

(4) $$r_{1}=\left(\frac{Q_{1}}{Q_{2}}\right)e^{-e_{1}(e_{1}-e_{2})}$$

(5) $$r_{2}=\left(\frac{Q_{2}}{Q_{1}}\right)e^{-e_{1}(e_{1}-e_{2})}$$

In these, *Q*_1_ and *Q*_2_ are indicators of monomer reactivity related to the resonance stability of the monomer, and the constants e_1_ and e_2_ are indicators of monomer polarity.

This is due to their similar structural characteristics and reactivity, making them more likely to interact with each other during the reaction and form copolymer chains. This similarity promotes the reaction between GMA and St, thereby driving the progression of the macromolecular free radical copolymerization reaction, which makes GMA more likely to copolymerize with St macromolecular free radicals. Generally speaking, the use of co-graft monomers can significantly enhance the GD and GE of functional monomers on polymers [48,54,56,61,62]. This study used St as a co-graft monomer to graft GMA more efficiently onto SEBS, as St can activate the macromolecular chains of SEBS, providing reactive sites for GMA to graft onto SEBS [32,33,58,63]. The reactivity of St with macromolecular free radicals is greater than that of GMA, so St preferentially reacts with the SEBS molecular chain, forming SEBS-g-St, and then reacts with GMA, thereby increasing the GD and GE of the graft product [64,65,66].

As shown in Table 3, GD and GE of GMA increased gradually with increasing GMA addition amount. This is because as the concentration of GMA in the reaction system increases, the probability of macro radicals generated by DCP initiating agent reacting with GMA is higher, thus improving the grafting degree and efficiency of GMA [67]. It can also be seen from Table 3 that when the GMA addition amount in the graft copolymer was 5%, the GE of GMA decreased slightly. This is because with increasing GMA amount in the graft copolymer, when the grafting extent reaches a certain level, the grafting sites at the chain ends of the main chain polymer become saturated and cannot undergo further grafting reactions with more excess added GMA. At this point, the large amount of unreacted GMA monomers will generate oligomer species through free radical polymerization among themselves, rather than participating in the grafting process onto the main chain. In addition, since the amount of initiator used is fixed, the number of macro free radicals that can be generated by the reaction system per unit time is also limited. Excessive GMA cannot react with the active centers within the effective time; thus, side reactions like self-polymerization and copolymerization occur. Therefore, the grafting efficiency decreases when the GMA content is too high in the SEBS-g-(GMA-co-St) graft copolymer [42].

### 3.3. DMA Analysis

**Figure 4:**
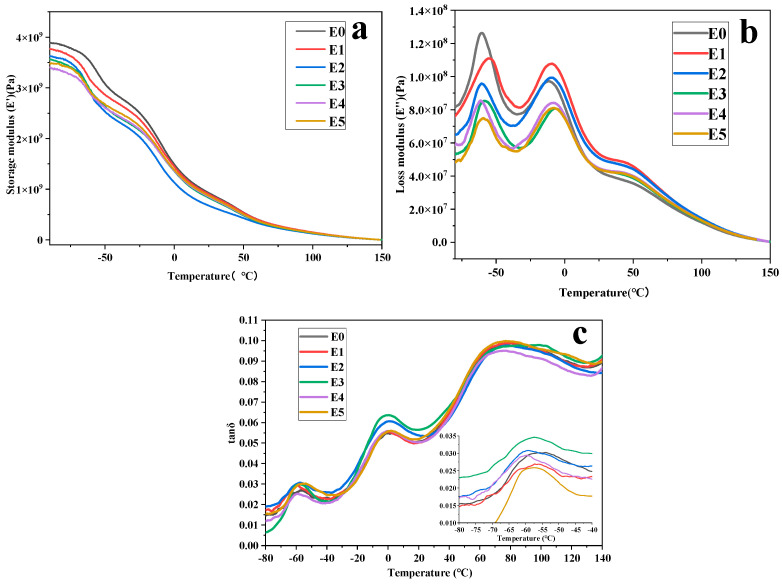
Dynamic mechanical analysis. (**a**) Storage modulus, (**b**) loss modulus, and (**c**) tan δ of PP/SEBS (E0) and the PP/SEBS/SEBS-g-(GMA-co-St) blends with different GMA amount: (E1) 1 wt%, (E2) 2 wt%, (E3) 3 wt%, (E4) 4 wt%, (E5) 5 wt%.

DMA can influence the storage modulus (E’), loss modulus (E’’), and damping loss factor (tan δ) of composite materials, including factors such as frequency and temperature [35]. The test results are shown in Figure 4. Figure 4a displays the E’-temperature curves of PP/SEBS and PP/SEBS/SEBS-g-(GMA-co-St) blends. In the low-temperature region, all samples exhibit a glassy state plateau, then transition to a rubbery state. It can also be observed from Figure 4a that within the studied temperature range, the E’ of each blend continuously decreases as the experimental temperature increases. Moreover, the E’ of the PP/SEBS/SEBS-g-(GMA-co-St) blend reveals a declining trend compared to the pure PP/SEBS blend, indicating that the internal frictional resistance between the large molecular chains in the system has decreased.

As shown in Figure 4b, all blends exhibit three peak values of E’’ within the test temperature range. These peaks correspond to the T_g_ of SEBS (olefin block), PP, and SEBS (styrene block) [68]. As can be seen from Figure 4c, three distinct relaxation peaks can be defined in the tan δ curve of the samples, corresponding one-to-one with the peaks described in Figure 4b. However, there is a significant deviation between the peak values of the T_g_ of SEBS (styrene block) in the tan δ curve and the E’’ curve. This mechanism is believed to originate from the relaxation and turning motion of the amorphous side chains of the PP material. As the GMA molecular chain increases, the internal friction increases, and the rigidity of the styrene segment in the SEBS large molecular chain causes the composite material to relax towards a high temperature [69].

Table 5 lists the corresponding data, where ΔT_g_ is obtained by subtracting the T_g_ corresponding to PP from the average temperature corresponding to the two relaxation peaks belonging to SEBS. Overall, after introducing SEBS-g-(GMA-co-St) into the PP/SEBS blend, as the amount of GMA added to the system increases, the two gradually converge, indicating that SEBS-g-(GMA-co-St) has a good compatibilizing effect on the PP/SEBS blend. When the GMA addition amount is 1 wt%, the ΔT_g_ of the blend, compared to the PP/SEBS blend without added SEBS-g-(GMA-co-St), decreases by 2.69 °C. Further increasing the GMA amount, the ΔT_g_ of the blend further decreases. When the GMA amount is 3 wt%, the ΔT_g_ of the blend drops to the lowest (7.76 °C), a decrease of 4.29 °C compared to the blend without the introduction of SEBS-g-(GMA-co-St). At this point, the compatibility between PP and SEBS phases is the best, and the introduction of the graft copolymer SEBS-g-(GMA-co-St) enhances the compatibility between the PP and SEBS phases [70].

### 3.4. DSC Analysis

**Figure 5:**
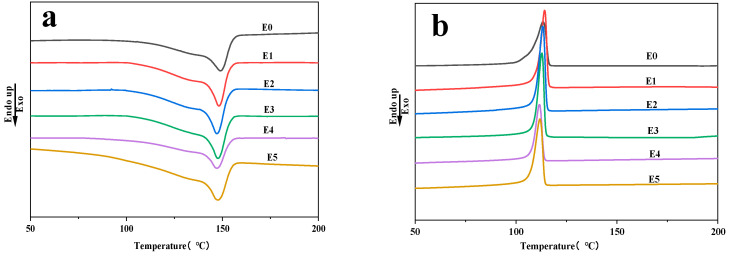
DSC curves. (**a**) Second heating scan and (**b**) cooling scan of PP/SEBS (E0) and the PP/SEBS/SEBS-g-(GMA-co-St) blends with different GMA amounts: (E1) 1 wt%, (E2) 2 wt%, (E3) 3 wt%, (E4) 4 wt%, (E5) 5 wt%.

Figure 5 presents the crystallization and second heating curves for the PP/SEBS and PP/SEBS/SEBS-g-(GMA-co-St) blends, while Table 6 lists the specific thermal parameters for the blends. As seen from Figure 5a, the melting temperature (T_m_) of the PP/SEBS blend is 149.74 °C. However, with the introduction of the SEBS-g-(GMA-co-St) graft copolymer, the T_m_ of the PP/SEBS/SEBS-g-(GMA-co-St) blend system shows a general downward trend. This is because the incorporation of the SEBS-g-(GMA-co-St) graft copolymer can reduce the regularity of the system’s chain segments, which in the microscopic view of the melting process means that lower energy is required to demonstrate melting behavior. Thus, a decreasing trend in T_m_ is displayed in the DSC curves.

As shown in Figure 5b and Table 6, the crystallization temperature (T_c_) of the PP/SEBS blend was 114.32 °C. However, the Tc of the PP/SEBS/SEBS-g-(GMA-co-St) blend system increases slightly, and with the increasing amount of GMA in the graft copolymer, both the T_c_ and the degree of crystallinity (X_c_) of the blend gradually decrease. The inclusion of the graft copolymer acts as a bridge between PP/SEBS, directly reducing the migration rate of large PP molecular chains and inhibiting the movement and crystallization arrangement of PP chain segments. With the increase in the amount of GMA added in the graft copolymer, more active sites can be introduced into the PP/SEBS blend system. This further enhances the structural hindrance effect between PP and SEBS, thereby slightly reducing the crystallization ability of the blend system [71,72,73].

### 3.5. WAXD Analysis

**Figure 6:**
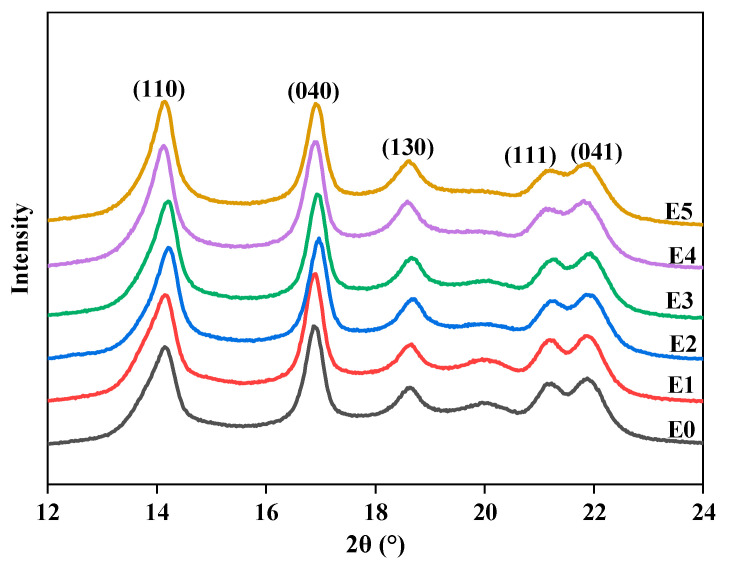
WAXD curves of PP/SEBS (E0) and the PP/SEBS/SEBS-g-(GMA-co-St) blends with different GMA amounts: (E1) 1 wt%, (E2) 2 wt%, (E3) 3 wt%, (E4) 4 wt%, (E5) 5 wt%.

The WAXD of PP/SEBS and PP/SEBS/SEBS-g-(GMA-co-St) blends is shown in Figure 6, with the corresponding 2θ data presented in Table 7. From Figure 6 and Table 7, it can be observed that both PP/SEBS and PP/SEBS/SEBS-g-(GMA-co-St) blends exhibit characteristic diffraction peaks at 2θ = 14.1°, 16.9°, 18.6°, 21.2° and 21.8°, which correspond to the (110), (040), (130), (111) and (041) planes of PP crystals, respectively [74]. Moreover, it can also be noticed from the figure that no new diffraction peaks appeared in the blend, which clearly indicates that the introduction of the graft copolymer does not alter the crystal structure of PP [75].

The grain size of the blends can be calculated using the Scherrer equation [76] as shown in Equation (6), with the specific data presented in Table 7.

(6) $$D=\frac{K\lambda }{\beta cos\;\theta }$$

where *D* represents the average grain size, *K* is the Scherrer constant (typically around 0.89), *λ* is the wavelength of the X-ray used, *β* is the full width at half maximum (FWHM) of the diffraction peak, and *θ* is the Bragg angle.

Based on the data in Table 7, the average grain size of the blend increased from 10.24 nm to 11.37 nm. This is due to the introduction of the graft copolymer SEBS-g-(GMA-co-St), where GMA reacts with the two phases, connecting PP and SEBS. Consequently, the migration rate of PP molecules in the blend decreases, resulting in larger crystal nuclei and hence, in an increased crystal size. Simultaneously, the presence of the graft inhibits the effective stacking of PP molecules, leading to a decrease in crystallinity [77]. The combined effect of these factors results in an increase in the grain size of the blend and a reduction in crystallinity.

### 3.6. TGA Analysis

**Figure 7:**
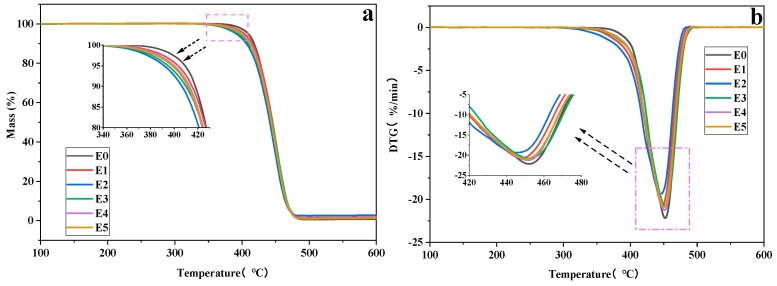
Thermogravimetric curves ((**a**) TG curves, (**b**) DTG curves)) of PP/SEBS (E0) and the PP/SEBS/SEBS-g-(GMA-co-St) blends with different GMA amounts: (E1) 1 wt%, (E2) 2 wt%, (E3) 3 wt%, (E4) 4 wt%, (E5) 5 wt%.

Figure 7 presents the TGA and DTG curves of the PP/SEBS and PP/SEBS/SEBS-g-(GMA-co-St) blends under a nitrogen atmosphere, and Table 8 lists the relevant parameters extracted from them. From the TGA curve (Figure 7a) and the data in Table 8, it can be seen that the initial decomposition temperature (T_95%_) of pure PP/SEBS is 409.23 °C, the maximum decomposition temperature (T_max_) is 451.66 °C, and the temperature at which 95 wt% of the weight loss (T_5%_) occurs is 471.49 °C. In comparison to pure PP/SEBS, the introduction of the SEBS-g-(GMA-co-St) graft copolymer decreases the T_95%_ of the blend system. When the GMA amount in the SEBS-g-(GMA-co-St) graft copolymer is 3 wt%, the T_5%_ of the PP/SEBS/SEBS-g-(GMA-co-St) blend decreases to 394.89 °C, a reduction of 14.34 °C compared to pure PP/SEBS. The decrease in thermal stability may be due to the increased complexity of the blend system with the introduction of the SEBS-g-(GMA-co-St) graft copolymer, leading to new chemical reactions, such as crosslinking or degradation reactions. These reactions might commence at lower temperatures, thereby reducing the thermal stability of the blend system. The decomposition temperatures of the grafted GMA and St components are generally lower than those of SEBS and PP, which may cause the SEBS-g-(GMA-co-St) graft copolymer to start decomposing earlier than the other two components during the heating process. Further increases in the GMA amount of the graft copolymer do not result in significant changes to the blend system’s T_5%_, which remains stable at around 395 °C. The introduction of the SEBS-g-(GMA-co-St) graft copolymer also does not have a significant impact on the blend system’s T_max_ and T_95%_, which remain stable at around 450 °C and 470 °C, respectively. Although GMA monomer itself has poor thermal stability, since it is present in low contents in the blend, only as trace grafts, its influence on the overall thermal performance of the blend is limited compared to the main polymers (PP, SEBS, etc.). In addition, with increasing GMA addition amounts in the SEBS-g-(GMA-co-St) graft copolymer, the grafting reaction between the SEBS segments and GMA will gradually become saturated. At this point, the excess GMA no longer participates in the grafting reaction, but exists as free monomers, whose negative impact on the thermal performance of the blend can be neglected.

### 3.7. Vicat Softening Temperature Testing

**Figure 8:**
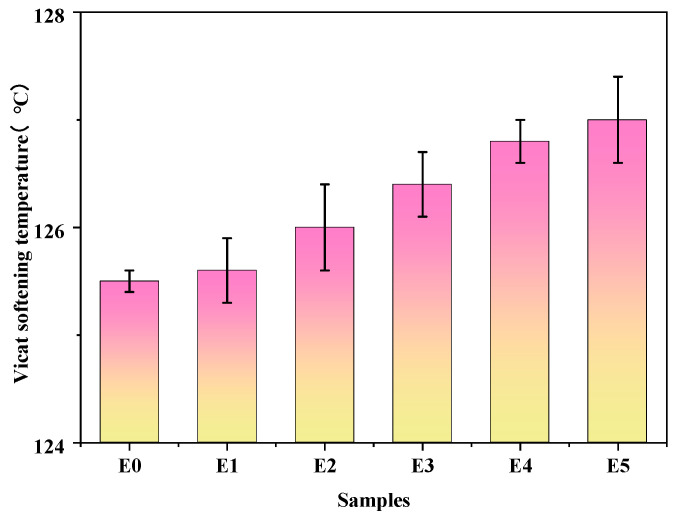
Vicat softening temperature of PP/SEBS (E0) and the PP/SEBS/SEBS-g-(GMA-co-St) blends with different GMA amounts: (E1) 1 wt%, (E2) 2 wt%, (E3) 3 wt%, (E4) 4 wt%, (E5) 5 wt%.

Figure 8 is a bar graph representing the effect of variations in GMA amount in the graft copolymer on the Vicat softening temperature of the PP/SEBS/SEBS-g-(GMA-co-St) blend. As can be seen from Figure 8, as the amount of GMA amount to the graft copolymer increases, the Vicat softening temperature of the blend gradually increases. This is due to the epoxy groups in GMA that can chemically react with the PP/SEBS molecular chains under heating conditions [78], forming a cross-linked structure or chemical bonds. SEBS is composed of both elastomeric (containing butadiene segments) and rigid (containing polystyrene segments) components, and has polar characteristics. In contrast, PP, due to its nonpolar properties [79], finds it difficult to interact with SEBS. With the introduction of GMA, the graft copolymer can bind with the rigid components in the SEBS molecules [80], enhancing the compatibility between SEBS and PP. This promotes their interaction and further raises the Vicat softening temperature of the blend.

### 3.8. MFR Analysis

**Figure 9:**
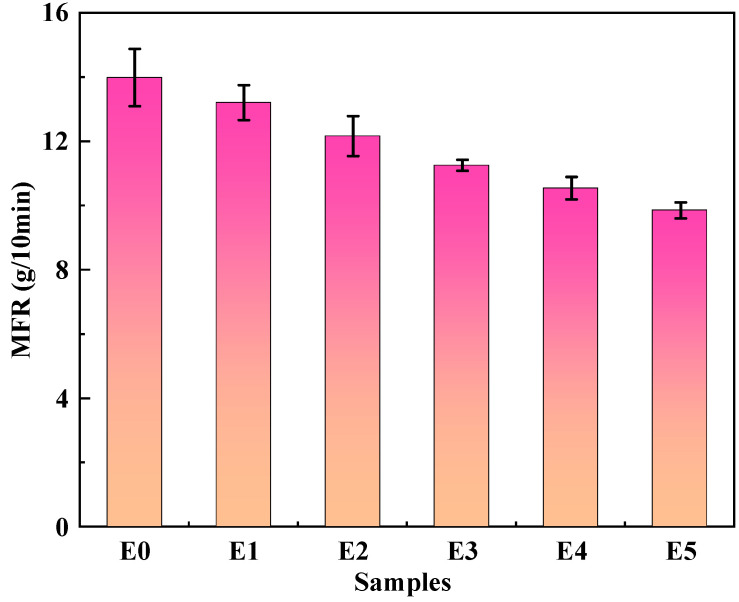
MFR curves of PP/SEBS (E0) and the PP/SEBS/SEBS-g-(GMA-co-St) blends with different GMA amounts: (E1) 1 wt%, (E2) 2 wt%, (E3) 3 wt%, (E4) 4 wt%, (E5) 5 wt%.

Figure 9 shows the melt flow rate of the PP/SEBS/SEBS-g-(GMA-co-St) blend as a function of the changing GMA amount in the graft copolymer, with Table 9 listing the relevant data. As can be seen from Figure 9, the MFR of pure PP/SEBS is 13.99 g/10 min. When 2 wt% GMA graft copolymer is added, the MFR of the blend starts to decrease (12.17 g/10 min). As the GMA addition amount in the graft copolymer continuously increases, the MFR value of the blends gradually decreases. When the GMA addition is 5 wt%, the MFR of the blend is 9.86 g/10 min, decreased by 3.35 g/10 min compared to pure PP/SEBS. This is because as more graft copolymer is introduced into the blend, the degree of crosslinking reactions in the system increases, which enhances the viscosity at the interface, further reducing the flowability of the blend [81].

### 3.9. Rheological Property Analysis

**Figure 10:**
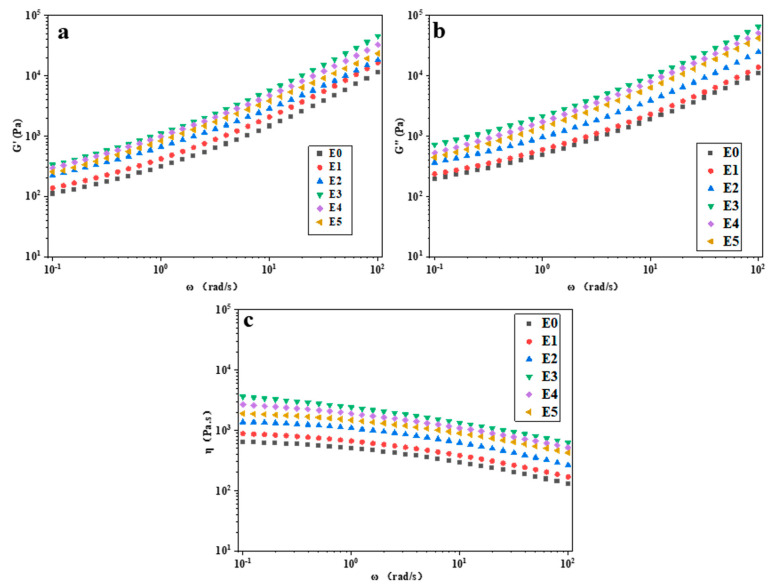
Rheological curves: (**a**) storage modulus, (**b**) loss modulus, (**c**) complex viscosity) of PP/SEBS (E0) and the PP/SEBS/SEBS-g-(GMA-co-St) with different GMA amounts: (E1) 1 wt%, (E2) 2 wt%, (E3) 3 wt%, (E4) 4 wt%, (E5) 5 wt%.

Figure 10 presents the storage modulus (G’), loss modulus (G″), and complex viscosity (η) of PP/SEBS and PP/SEBS/SEBS-g-(GMA-co-St) blends as functions of angular frequency ω (0.1–100 rad/s). As can be seen from Figure 10a,b, with the increase in GMA amount in the graft copolymer, the G’ and G″ of the blend exhibit a trend of first increasing and then decreasing. This is because the introduction of the graft copolymer causes different degrees of epoxidation characteristics in the blend system, thereby enhancing the entanglement ability of PP and SEBS segments. In addition, as the GMA amount in the graft copolymer increases, the degree of grafting of the SEBS-g-(GMA-co-St) graft copolymer also gradually increases, thereby enhancing the interaction between PP and SEBS, promoting the stability of their local structures, and thus increasing the G’ and G″ of the blend. However, the further increase in GMA amount in the graft copolymer (3–5 wt%) may lead to an increase in cross-linking reactions between side chains, thereby forming more cross-linking points. These crosslinking points restrict the movement of the molecular chains, leading to a decrease in dynamic modulus, and therefore the G’ and G″ of the blend decrease. As can be seen from Figure 10c, the η of each formulation decreases with increasing frequency, reflecting the shear thinning phenomenon, showing typical pseudoplastic fluid motion characteristics [73,82]. As the shear frequency increases, the entanglement between molecular chains in the blend decreases, thereby making the blend more fluid, which makes the PP/SEBS blend system exhibit more significant shear thinning behavior. In addition, in the low shear frequency region, the frictional resistance between the molecular segments of the blend is high, making it difficult for the molecular segments to flow. As the shear frequency increases, the frictional resistance between the molecular segments gradually decreases, and the interaction between the molecular segments weakens, so the η of the blend significantly decreases.

### 3.10. Optical Property Analysis

**Figure 11:**
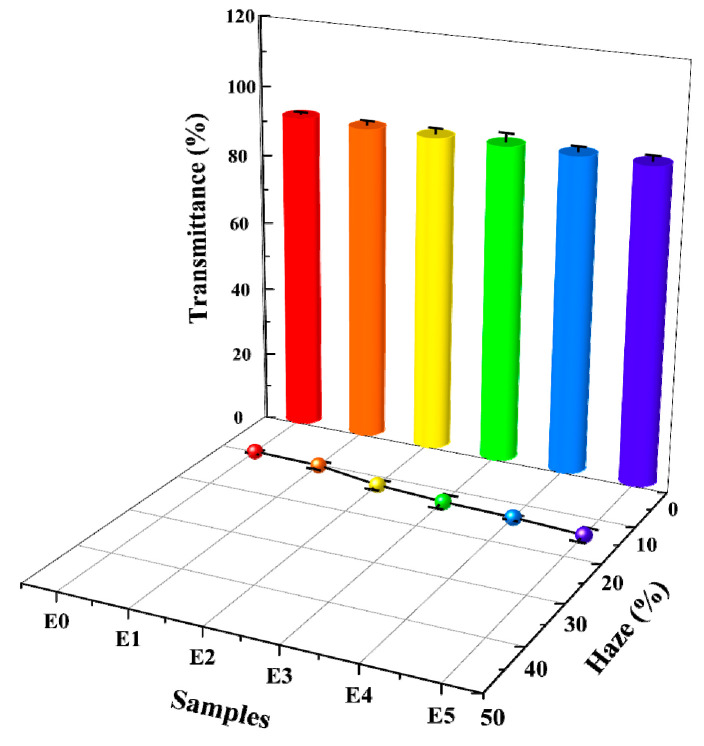
Haze and transmittance of PP/SEBS (E0) and the SEBS-g-(GMA-co-St) blends with different GMA amounts: (E1) 1 wt%, (E2) 2 wt%, (E3) 3 wt%, (E4) 4 wt%, (E5) 5 wt%.

This experiment discusses the effect of the amount of GMA added to the graft copolymer on the haze and transmittance of the PP/SEBS/SEBS-g-(GMA-co-St) blend system, as shown in Figure 11. The specific data can be found in Table 10. From Figure 11 and Table 10, it can be observed that with an increase in the amount of GMA added to the graft copolymer, there is a slight increase in the haze of the blend. This is mainly influenced by the crystallinity of the polymers, as generally, larger crystal sizes within the blend result in higher haze [83]. The WAXD analysis mentioned above also clearly demonstrates that the introduction of copolymer monomers in the SEBS-g-(GMA-co-St) graft copolymer leads to an increase in the crystal size of the blend, thereby resulting in higher haze. However, the transmittance of the blend fluctuates within a very small range (90.9–91.9%) when different amounts of GMA are introduced into the blend. This indicates that the SEBS-g-(GMA-co-St) graft copolymer has a minimal impact on the optical properties of the PP/SEBS/SEBS-g-(GMA-co-St) blend, thus maintaining the excellent optical performance of the material.

### 3.11. Mechanical Properties

**Figure 12:**
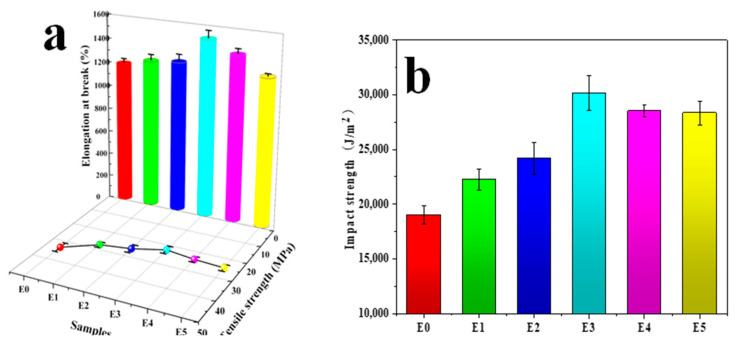
Effect of GMA amount on mechanical properties of PP/SEBS/SEBS-g-(GMA-co-St) blends. (**a**) Effect of GMA amount on the tensile properties of PP/SEBS/SEBS-g-(GMA-co-St); (**b**) effect of GMA amount on the impact properties of PP/SEBS/SEBS-g-(GMA-co-St). PP/SEBS (E0) (the red line) and the SEBS-g-(GMA-co-St) blends with different GMA amounts: (E1) 1 wt% (the green line), (E2) 2 wt% (the blue line), (E3) 3 wt% (the cyan line), (E4) 4 wt% (the purple line), (E5) 5 wt% (the yellow line).

**Figure 13:**
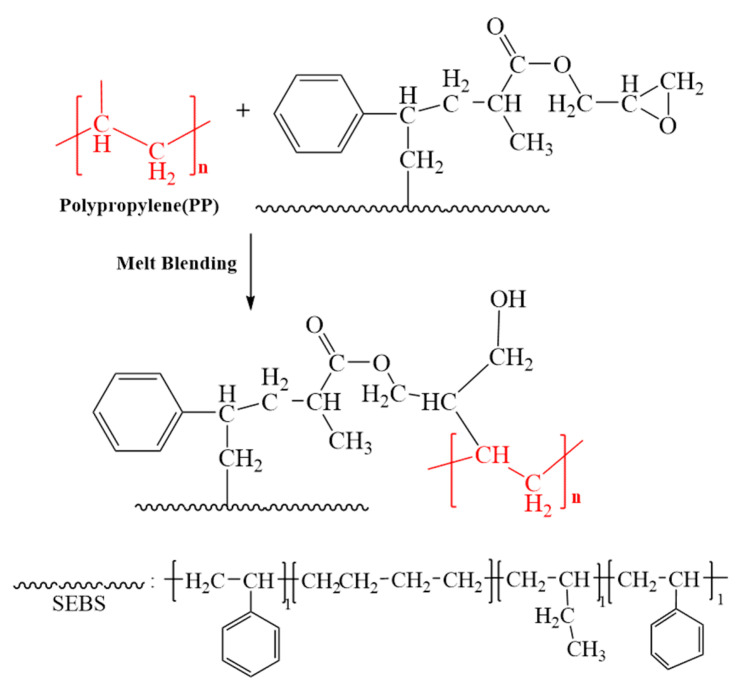
Reaction scheme for the compatibilization of SEBS-g-(GMA-co-St) graft copolymer(the red color is the PP added).

**Figure 14:**
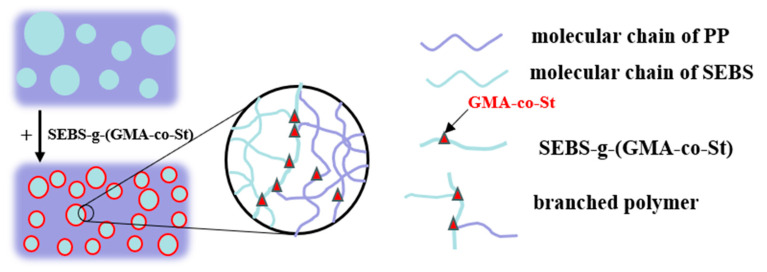
Schematic illustration of the reaction mechanism in PP/SEBS/SEBS-g-(GMA-co-St)(the red color is GMA-co-St).

Generally speaking, enhancing the compatibility between PP and SEBS is beneficial for improving the mechanical properties of the PP/SEBS blend [84]. Therefore, this study introduced SEBS-g-(GMA-co-St) graft copolymers into the PP/SEBS blend system to improve the compatibility between PP and SEBS and thereby enhance the blend’s mechanical properties. This study also examined the impact of the GMA amount in the graft copolymers on the impact strength and tensile properties of the PP/SEBS/SEBS-g-(GMA-co-St) blend. The specific effects are shown in Figure 12, and the specific data data are presented in Table 11. As can be seen from Figure 12 and Table 11, the introduction of SEBS-g-(GMA-co-St) graft copolymers into the PP/SEBS blend results in a slight decrease in the tensile strength of the PP/SEBS/SEBS-g-(GMA-co-St) blend. However, the fracture elongation and notched impact strength both significantly increase. The fracture elongation and notched impact strength both show a trend of first increasing and then decreasing with the increase of GMA amount in the graft copolymers. When the GMA amount in the graft copolymer is 3 wt%, the blend exhibits the best toughness, with a notched impact strength and fracture elongation reaching 30,165.82 J/m^2^ and 1445.32%, respectively. The underlying reason for these phenomena is that the increase in the GMA amount in the graft copolymer leads to more epoxy groups reacting with the end groups of PP/SEBS, thereby significantly improving the compatibility between the two phases. Consequently, the interfacial area can better transmit and bear stress, thus gradually increasing the impact performance and fracture elongation of the blend. The reaction route is depicted in Figure 13, and a schematic of the SEBS-g-(GMA-co-St) graft copolymer compatibilizing the PP/SEBS blend is shown in Figure 14. However, when the GMA amount in the graft copolymer exceeds 3 wt%, the blend’s toughness starts to decline. This may be due to the fact that the excess epoxy groups in the graft copolymer begin to react with each other, leading to cross-linking side reactions. These reactions create cross-linked networks in the blend, which hinder the movement of the polymer chains and negatively affect the blend’s ductility, leading to a decrease in impact strength and fracture elongation [85].

### 3.12. SEM Analysis

**Figure 15:**
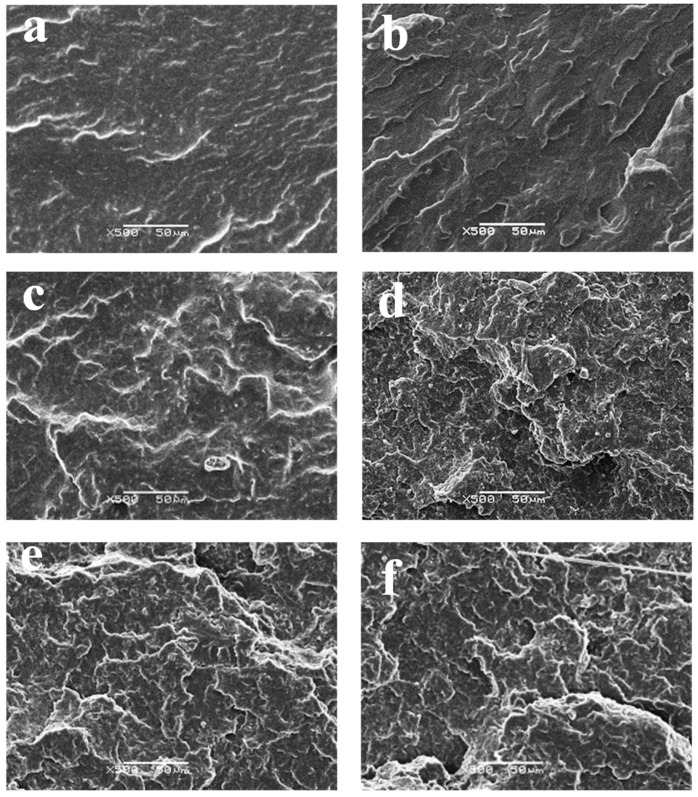
Microstructure of impact fracture surface of PP/SEBS (**a**) and the PP/SEBS/SEBS-g-(GMA-co-St) blends with different GMA amounts: (**b**) 1 wt%, (**c**) 2 wt%, (**d**) 3 wt%, (**e**) 4 wt%, (**f**) 5 wt%.

To investigate the variation in fracture morphology of PP/SEBS/SEBS-g-(GMA-co-St) blends with the change in GMA amount in the graft copolymer, SEM was used to observe the impact fracture surface of the blend, as shown in Figure 15. As can be seen from Figure 15a, the fracture surface of PP/SEBS is relatively smooth. After introducing the SEBS-g-(GMA-co-St) graft copolymer into the PP/SEBS blend, the fracture morphology of the blends is markedly different from that of PP/SEBS. When the GMA amount in the graft copolymer varies within the range of 1~2 wt%, as shown in Figure 15b,c, the roughness of the impact fracture surface of the blend increases. This is because the introduction of the graft copolymer effectively reduces the interfacial tension, increases the interfacial interaction between the two phases, thereby improving the impact toughness of the blend. Although the increase in impact strength of the blend is small, it can still be observed that the fracture surface becomes rougher and uneven, and wrinkles caused by crack branching during the fracture process can also be observed. As the GMA amount in the graft copolymer increases to 3 wt%, as shown in Figure 15d, the dispersion phase interface in the system adheres more tightly, and the impact fracture surface of the blend presents a high and low undulation morphology, showing a more obvious layered phenomenon. The roughness of the fracture surface also further increases, presenting a wrinkled toughness fracture characteristic. This indicates that at this time, the graft copolymer significantly enhances the toughness of the PP/SEBS blend, and more obvious plastic deformation occurs on its fracture surface, and it is precisely because of this deformation that the impact toughness of the blend greatly improves [86]. With further increases in the GMA amount in the graft copolymer (4~5 wt%), as shown in Figure 15e,f, the roughness of the impact fracture surface of the blend slightly decreases, and plastic deformations weaken. This is because when the GMA amount in the graft copolymer is too high, more cross-linking reactions will occur at the interface, and the large number of cross-linked structures will greatly restrict the movement of the interface, thereby reducing the impact performance of the blend [54]. In summary, the results of the microscopic morphology are consistent with the results of the mechanical performance test.

## 4. Conclusions

St was incorporated as a comonomer into a melt grafting system of GMA and SEBS, creating graft copolymers of SEBS-g-(GMA-co-St) with varying GMA amounts. Subsequently, the prepared graft copolymers were melt-blended with PP and SEBS, yielding a series of PP/SEBS/SEBS-g-(GMA-co-St) blends. Comprehensive studies were conducted on the thermal, optical, rheological, and mechanical properties, and microscopic morphology of these blends. The main findings are as follows:

(1) FTIR and ^1^H-NMR analyses demonstrate that GMA and St successfully graft onto SEBS to form SEBS-g-(GMA-co-St) graft copolymers. As the amount of GMA in the graft copolymer increases, the degree of GMA grafting gradually increases while the grafting efficiency remains relatively stable.

(2) Thermo-rheological and crystallographic analyses show that the G’, G″, and η of the blend consistently increase as the amount of GMA in the graft copolymer increases. The introduction of SEBS-g-(GMA-co-St) graft copolymers reduces the migration rate of large polymer chains, hindering the motion and crystalline arrangement of PP chains, consequently lowering the degree of crystallinity. DMA analysis reveals that when the GMA amount in the SEBS-g-(GMA-co-St) graft copolymer reaches 3 wt%, the ΔT_g_ between the PP and SEBS phases is minimized (7.76 °C), indicating optimal blend compatibility.

(3) From an optical perspective, as the GMA amount in the graft copolymer increases, the transmittance of the blend remains above 90.9%, while the haze of the blend system undergoes only slight changes. This indicates that the SEBS-g-(GMA-co-St) graft copolymer has a minimal impact on the optical properties of the PP/SEBS/SEBS-g-(GMA-co-St) blend, which still exhibits excellent optical performance.

(4) In terms of mechanical properties and microscopic morphology, within the examined GMA amount range (1–3 wt%), the notched impact strength and fracture elongation of the blend increase as the GMA amount in the graft copolymer increases. Specifically, when the GMA amount reaches 3 wt%, the blend exhibits the best toughness, with a notched impact strength and fracture elongation reaching 30,165.82 J/m^2^ and 1445.32%, respectively. At this point, the blend’s fracture mode manifests notable ductile fracture characteristics. Increasing the GMA amount in the graft copolymer beyond this point will induce a certain degree of cross-linking side reactions, deteriorating the performance of SEBS-g-(GMA-co-St), eventually disrupting the blend’s phase morphology and reducing its mechanical properties.

## Figures and Tables

**Table 1:** The recipe of the SEBS-g-(GMA-co-St) graft copolymers with different amount of GMA.

Samples	SEBS (wt%)	GMA (wt%)	St (wt%)	GMA/St (wt/wt)	DCP (wt%)
D1	100	1	2	1:2	0.3
D2	100	2	4	1:2	0.3
D3	100	3	6	1:2	0.3
D4	100	4	8	1:2	0.3
D5	100	5	10	1:2	0.3

**Table 2:** The recipe of the PP/SEBS/SEBS-g-(GMA-co-St) graft copolymers with different amount of GMA.

Samples	Blend Composition (wt%)
E0	PP/SEBS = 80/20
E1	PP/SEBS/D1 = 80/10/10
E2	PP/SEBS/D2 = 80/10/10
E3	PP/SEBS/D3 = 80/10/10
E4	PP/SEBS/D4 = 80/10/10
E5	PP/SEBS/D5 = 80/10/10

**Table 3:** Effect of GMA amount on GD and GE of SEBS-g-(GMA-co-St) graft copolymers.

Samples	GD (phr)	GE (%)
D1	0.41	41.0
D2	0.85	42.5
D3	1.35	45.0
D4	1.91	47.8
D5	2.21	44.2

**Table 4:** Q and e values of GMA and St.

	GMA	St
Q	0.98	1.00
e	0.20	−0.8

**Table 5:** Thermal properties characterization data of different samples via DMA.

Samples	T_g1_ (°C)	T_g2_ (°C)	T_g3_ (°C)	ΔT_g_ (°C)
E0	−54.96	2.89	84.83	12.05
E1	−57.24	1.12	78.22	9.36
E2	−57.06	1.62	78.35	9.03
E3	−58.71	0.91	76.06	7.76
E4	−56.70	2.52	79.34	8.79
E5	−56.85	1.83	79.13	9.31

**Table 6:** Thermal properties of different samples during cooling scan and second heating scan.

Samples	T_c_ (°C)	T_m_ (°C)	$∆H_{m}$ (J/g)	X_c_ (%)
E0	114.32	149.74	48.30	28.89
E1	115.06	148.43	58.45	34.96
E2	114.32	147.76	57.58	34.44
E3	114.18	147.42	55.87	33.42
E4	112.75	148.06	52.68	31.51
E5	112.29	147.56	48.62	29.08

**Table 7:** 2θ data and grain size of different samples.

	Samples	(110)	(040)	(130)	(111)	(041)	Average D (nm)
2θ (°)	E0	14.07	16.88	18.62	21.18	21.89	10.24
2θ (°)	E1	14.06	16.88	18.62	21.18	21.89	10.33
2θ (°)	E2	14.07	16.88	18.58	21.13	21.85	10.43
2θ (°)	E3	14.15	16.95	18.66	21.22	21.93	10.80
2θ (°)	E4	14.13	16.93	18.65	21.22	21.94	10.95
2θ (°)	E5	14.11	16.92	18.60	21.17	21.88	11.37

**Table 8:** Thermogravimetry data of different samples.

Samples	T_95%_ (°C)	T_max_ (°C)	T_5%_ (°C)
E0	409.23	451.66	471.49
E1	403.41	448.06	472.68
E2	391.55	446.39	470.98
E3	394.89	451.46	470.85
E4	400.20	451.39	471.50
E5	398.58	450.64	471.26

Note: T_95%_ is the temperature at which the sample loses 5% of its mass, T_max_ is the temperature at which the sample loses weight at its maximum rate, and T_5%_ is the temperature at which the sample loses 95% of its mass.

**Table 9:** MFR of PP/SEBS and PP/SEBS/SEBS-g-(GMA-co-St) blends.

Samples	MFR/(g/10 min)
E0	13.99 ± 0.89
E1	13.20 ± 0.54
E2	12.17 ± 0.62
E3	11.26 ± 0.17
E4	10.55 ± 0.35
E5	9.86 ± 0.25

**Table 10:** Optical performance data of PP/SEBS and PP/SEBS/SEBS-g-(GMA-co-St) blends.

Samples	Haze (%)	Transmittance (%)
E0	9.8 ± 0.6	91.9 ± 0.6
E1	10.2 ± 1.2	91.2 ± 1.2
E2	12.2 ± 1.4	91.1 ± 1.5
E3	13.1 ± 1.9	91.2 ± 2.5
E4	13.5 ± 0.7	91.0 ± 1.4
E5	14.3 ± 1.8	90.9 ± 1.5

**Table 11:** Mechanical property data of PP/SEBS and PP/SEBS/SEBS-g- (GMA-co-St) blends.

Samples	Impact Strength/(J/m^2^)	Elongation at Break/(%)	Tensile Strength/(MPa)
E0	19,022.21 ± 452.56	1200.6 ± 50.6	33.51 ± 3.34
E1	22,247.86 ± 983.35	1245.3 ± 47.2	27.66 ± 1.52
E2	24,160.47 ± 1484.90	1268.7 ± 55.8	25.98 ± 2.22
E3	30,165.82 ± 1560.57	1445.3 ± 62.6	22.52 ± 2.1
E4	28,531.64 ± 578.25	1388.9 ± 40.5	23.99 ± 1.56
E5	28,347.34 ± 1083.64	1241.7 ± 20.9	24.73 ± 1.74

## Data Availability

The data presented in this study are available on request from the corresponding author.
